# (-)-α-Bisabolol inhibits D-Gal-induced HSF cellular senescence *in vitro* and prevents skin aging *in vivo* by reducing SASP

**DOI:** 10.22038/IJBMS.2024.76073.16469

**Published:** 2024

**Authors:** Meixing He, Panyu Zhou, Hankun Chen, Junhong Zhang, Yating Zhang, Xianhui Zheng, Wei Zhu, Ling Han

**Affiliations:** 1 The Second Clinical College, Guangzhou University of Chinese Medicine, Guangzhou, China; 2 Guangdong Provincial Hospital of Traditional Chinese Medicine, Guangzhou, China; 3 Research and Development Department, Guangzhou Qinglan Biotechnology Company Limited, Guangzhou, China; 4 State Key Laboratory of Dampness Syndrome of Chinese Medicine, The Second Affiliated Hospital of Guangzhou University of ChineseMedicine, Guangzhou, China; 5 Guangdong Academy of Traditional Chinese Medicine, Research Team of Bio-molecular and System Biology of Chinese Medicine, Guangzhou, China; 6 Guangdong Provincial Key Laboratory of Clinical Research on Traditional Chinese Medicine Syndrome, Guangzhou, China; 7 Guangdong-Hong Kong-Macau Joint Lab on Chinese Medicine and Immune Disease Research, Guangzhou University of Chinese Medicine, Guangzhou, China

**Keywords:** (-)-α-Bisabolol, Cellular senescence, D-Galactose, SASP, Skin aging

## Abstract

**Objective(s):**

To study the anti-aging effect of (-)-α-bisabolol ((-)-α-bis) on the skin and preliminarily clarify its mechanism.

**Materials and Methods:**

Human skin fibroblasts (HSF) were induced senescence by D-Galactose. Senescence β-galactosidase staining was utilized to evaluate the senescence of HSF. TNF-α, IL-6, IL-8, IL-1β, CCL-2, CCL-5, and MMP-9 in senescence-as-sociated secretory phenotype (SASP) were detected by RT-qPCR. Meanwhile, aged BALB/c mice were applied topically with 0.5% and 2%(-)-α-bis gel for 30 days continuously to evaluate anti-aging parameters on the skin such as surface measurement, the Trans Epidermal Water Loss (TEWL), and skin barrier index of dorsal skin. Then, HE staining, Masson staining, and IHC were applied to measure epidermal thickness, collagen fiber content in the dermis, and content of dermal collagen I, respectively. Last, SOD, MDA, and HYP contents of the back skin tissue of mice were also detected.

**Results:**

(-)-α-Bis reduced the expression of senescence-associated β-galactosidase (SA-β-gal) and expression levels of SASP in HSF cells stimulated by D-Gal (*P*<0.05). Mice aged 9 months were applied locally with (-)-α-bis gel to improve skin aging, the TEWL and skin barrier index of dorsal skin, and ameliorate the epidermal thickness and contents of dermal collagen fibers and collagen I (*P*<0.05). Furthermore, (-)-α-bis up-regulated the mRNA expression levels of elastin and collagen III effectively (*P*<0.05).

**Conclusion:**

(-)-α-Bis can delay the senescence of HSF cells by reducing the expression of SA-β-gal and SASP factors *in vitro*. Improved skin barrier function as well as SASP is responsible for the delay of skin aging *in vivo*.

## Introduction

Decrepitude of skin is a direct manifestation of body aging, and naturally aging skin will appear with wrinkles, inelasticity, roughness, and other symptoms (1). Aging skin is not only unfriendly for appearance, it also leads to disorders of skin function and even increases the risk of skin diseases such as skin cancer (2). At present, popular theories of skin aging primarily include radical scavenging, oxidative stress injury, mitochondrial DNA damage, etc. (3). DNA damage caused by chemotherapy or ultraviolet irradiation can lead to increased expression of senescence-associated β-galactosidase (SA-β-gal) and the secretion of senescence-as-sociated secretory phenotype (SASP) factors such as inflammatory cytokines (TNF-α, IL-6, IL-8, and IL-1β), chemokines (CCL-2 and CCL-5), and MMPs (MMP-2, MMP-3, and MMP-9). DNA damage reaction also brings about the up-regulation of cell cycle arrest proteins p53, p21, and p16. D-Gal-induced cellular and animal models are internationally recognized models of aging and have been extensively applied in the realm of anti-decrepitude drugs (4). Currently, western medicine mainly adopts surgical operations, local injection of kreotoxin, subcutaneous injection of collagen, and other methods to tighten the skin, combined with anti-oxidants to treat skin aging, which has certain side effects. Small molecular compounds that resist aging possess naturally low toxicity and outstanding effects, which can treat skin aging.

(-)-α-Bis is a kind of monocyclic sesquiterpene alcohol, which exists in *chamomilia*, *myrrha*, *moslae herba,* and other plants. Due to its anti-oxidant, anti-inflammatory, and anti-apoptotic activities, several international researchers have reported a variety of biological activities in pancreatic cancer cells and other cancer cell lines, including potential therapeutic effects (5-7). For example, α-bis has recently been reported to inhibit human non-small cell lung carcinoma cells by apoptosis induction, cell cycle arrest, anti-migration, and anti-invasion (8). Recently, the researchers concluded that α-bis microspheres can scavenge ABTS(•) and DPPH(•) free radicals with α-bis prepared into TiO_2_ microspheres (9). In general, senescent cells develop anomalous morphology, such as increased volume, enhancive granules, and obvious flattening. SA-β-gal is the earliest and most widely used biomarker of aging, which is still the golden standard for the identification of decrepitude cells and tissues *in vitro*. The decrease of elastin and collagen are also considered to be the main signs of skin aging (10). Reversing the expression of genes and proteins mentioned above has been reckoned as a rational strategy to prevent skin aging (10, 11). The dermis is mainly composed of fibroblasts, while the decrease in the number, the change of morphology, and the degeneration of secretion and synthesis functions of fibroblasts are closely related to skin aging. In this study, human skin fibroblasts (HSF) are used to simulate the aging state of human dermis fibroblasts. (-)-α-Bis has been used in multiple skincare products, but its pharmacological effect on aging skin is still absent. In this research, the anti-aging effect of (-)-α-bis and its molecular mechanism are conducted.

In this study, D-Gal-stimulated HSF cells and naturally aged BALB/c mice are used to explore the anti-aging effects of (-)-α-bis as aging phenotypes and SASP genes are investigated.

## Materials and Methods

***Chemicals and antibodies***

(-)-α-bis (CAS : 23089-26-1, 95% purity) was purchased from Baoji Herbest Bio-Tech Co., Ltd. HSF cells were derived from ICell Bioscience Inc. Dulbecco’s Modified Eagle’s Medium (DMEM) was obtained from GIBCO^®^ Invitrogen (Auckland, New Zealand). Anti-human p16 antibody was offered from Cell Signaling Technology. Anti-human p21 antibody was received from Abcam’s official website. Collagen I antibody was gained from Boster Biological Technology Co. Ltd. Senescence β-Galactosidase Staining Kit was bought from Beyotime Biotechnology. Primer TNF-α, IL-6, IL-8, etc., were acquired from Thermo Fisher Scientific. Superoxide dismutase (SOD) kit, malondialdehyde (MDA) kit, and hydroxyproline (HYP) kit were gotten from Nanjing Jianguo Bioengineering Institute (China).

***Cell culture and D-Gal stimulation***

HSF cells were cultured in DMEM with 10% fetal bovine serum, 100 U/ml penicillin, and 100 μg/ml streptomycin in a 37 °C, 5% CO_2 _incubator. Cells in the D-Gal group were only exposed to 40 mg/ml D-Gal for 24 hr. In the (-)-α-bis groups, cells were pretreated with (-)-α-bis of 25 μM, 50 μM, and 100 μM 12 hr, respectively, then they were exposed to medium including 40 mg/ml D-Gal and the (-)-α-bis of corresponding concentrations for another 24 hr. Cells in the control group were cultured with a normal medium while the positive drug group was treated with 50 μg/ml vitamin C (VC) (12).

***MTT assay***

Cell viability was determined by the MTT method. In simple terms, HSF cells (1 × 10^5 ^/well) were seeded into 96-well plates. After administration, we added 100 μl MTT solution of 5 mg/ml to each well, and then cells were incubated in the darkness for 4 hr. Culture medium was discarded, and we dissolved the crystals with DMSO of 100 μl. The absorbance was detected at 570 nm using an automatic microplate reader (Tecan M1000 PRO).

***SA-β-gal activity assay in vitro***

SA-β-gal activity in HSF cells was analyzed to determine the down-regulation effect according to the kit instructions. After 1 × 10^4^ cells were seeded in a 6-well plate, we allowed them to adhere for 12 hr before administering 40 mg/ml D-Gal for 24 hr for stimulation. After the plate was collected, the medium was disposed and the wells were rinsed with PBS buffer, at last, the reagent prepared in accordance with the instructions was added to the wells. The morphology and the number of HSF cells stained with SA-β-gal kit were observed with a light microscope (× 200) after 12 hr incubation at 37 °C without CO_2_ and shielded from light. Three different fields were randomly selected as the number of blue-stained cells and the samples were compared with the mod group. Data are mean ± SD of independent experiments (*n=* 3, each group). Compared with the mod group, *P<0.05, **P<0.01, and ***P<0.001.

***Real-time quantitative PCR(RT-qPCR)***

For overall RNA extraction, HSF cells were maintained with (-)-α-bis for 12 hr before exposure to 40 mg/ml D-Gal for 24 hr. After 24 hr, the overall RNA was extracted with Trizol lysate from Accurate Biology’s official website. Purified 1 μg total RNA was reversed-transcribed by making full use of EZB’s reverse transcription reagent (EZBioscience) and amplified using AG’s amplification reagent (Accurate Biology). Amplification conditions included 40 cycles of denaturation at 95 °C for 30 sec, annealing at 95 °C for 05 sec, and finally amplification at 60 °C for 30 sec in ViiA^TM^ 7 thermal cycler (Bio-Rad Laboratories). The primer sequences adopted are as follows (Table 1).

***Western blot analysis***

HSF cells (1 × 10^5^) were maintained in a 60 mm diameter culture dish for 24 hr to wait for attachment, preadministered in medium containing 10% serum for 12 hr, and then followed by exposure to 40 mg/ml D-Gal stimulation for 24 hr at the indicated time. To determine the expression levels of cyclin p16 and p21 in the cells, the collected cells were lysed with lysate and the protein concentration was measured by using a BCA protein detection kit (Thermo Fisher Scientific). Besides, the protein was boiled with a loading buffer for 5 min, as stated in the manufacturer’s manuals. Denatured proteins (25 μg) were subjected to 12% sodium dodecyl sulfate polyacrylamide gel electrophoresis (SDS-PAGE). Isolated proteins were transferred to 0.2 μm polyvinylidene fluoride membranes (Merck Millipore, USA). After blotting, the membranes were incubated with a specific primary antibody overnight at 4 °C. After hybridizing with a secondary antibody, protein bands were observed by ChemiDoc Touch using an ECL chemiluminescence hypersensitivity color development kit. GAPDH was used as loading control for p16 and p21.

***Experimental animals***

1.5-2 month (1.5–2 m) and 9 month old (9 m) male BALB/c mice (25±5g) were purchased from Guangdong Medical Laboratory Animal Center. The mice acclimated to the environment and had free access to food and water for a week before the experiment. All animals were reared in climate-controlled conditions (24 °C, 50% humidity) with a 12 hr light-dark cycle. Experiments were compliant with the regulations and approval of the Institutional Animal Care and Use Committee of Guangdong Hospital of Traditional Chinese Medicine (the ethical number: 2021089). A total of 36 BALB/c mice were randomly assigned to 6 groups (*n=* 6, per group) : 1.5–2 m control group (1.5–2 m con), 9 m untreated control group (9 m con), 9 m model vehicle group (9 m mod), positive drug vitamin E cream group (9 m VE), 9 m 0.5%(-)-α-bis gel (9 m low dose group), and 2%(-)-α-bis gel (9 m high dose group) (13). (-)-α-bis was mixed with a homemade gel containing glycerinum, carbomer, triethanolamine, and ultrapure water (refer to appendix). The gel was put into use externally for 30 days, twice a day. Vehicle gel was applied to the back of the 9 m mod group while no treatment was given to the 9 m con group, and vitamin E cream (1% concentration) was applied to the positive drug group. The Trans Epidermal Water Loss (TEWL) and the skin barrier index of the dorsal skin of mice were detected by a gpskin barrier function measurement instrument (gpskin) every 2 days. Before the end of the program, the experiment was stopped when the animals showed significant anxiety and restlessness, or the animals lost about 20% of their body weight. It was not until a month later that skin aging was observed in the 9 m mod group without causing pain to the animals. At the same time, the skin improvement of mice in administration groups was observed, and the experiment was completed by intraperitoneal injection of pentobarbital sodium with 100 mg/kg standard based on the body weight of mice.

***Preparation of skin tissue lysis***

Mice were anesthetized the day after the last dose, and the dorsal skin of each mouse was sheared. Adipose tissue was separated, and we instantly excised and homogenized the skin utilizing Trizol lysate, which was centrifuged at 13902g for 20 min at 4 °C. Then the RNA content in the supernatant was extracted with the extraction reagent. PCR analysis of elastin and collagen III gene levels was performed as described above. The primer sequences of elastin and collagen III are shown below (Table 1).

***Measurement of TEWL and skin barrier index***

TEWL and skin barrier index were measured by using the GPSkin (Beijing UnderProved Medical Technology Co., LTD). VE group, low dose group, and high dose group at 9 months of age compared to the 9 m mod group.

***Histological examination***

After shaving the fur with a sterile scalpel, 0.5 square centimeters skin on the back was soaked and fixed in 4% paraformaldehyde. Immediately the paraffin-embedded dorsal skin samples were cut into 4 μm thin sections, they would be stained with hematoxylin and eosin. Changes in epidermal thickness were measured and analyzed under a light microscope from the surface of the cuticle (SC) to the base of the dermal papilla.

***Masson staining***

After the 4 μm thin sections of skin tissues were dewaxed and dehydrated, the sections were stained successively with ponceau and aniline blue. After the sections were sealed, the changes in the morphology and number of collagen fibers in the dermis of the skin were observed with a light microscope. Three different visual fields were randomly selected as statistics of dermis thickness for each sample.

***Immunohistochemical staining***

After paraffin sections were deparaffinized and dehydrated, the skin tissues were deactivated with 3% hydrogen peroxide for 10 min, blocked using 5% normal goat serum for 1 hr, and then incubated applying primary antibody of collagen Ⅰ overnight. The primary antibody (1:800) was diluted with 5% normal goat serum. After blocking, the tissues were rinsed three times with PBST buffer and incubated with the rabbit secondary antibody for 30 min. The sections were washed again three times with PBST buffer. Antigens were detected using a 3,3N-Diaminobensidine Tetrahydrochloride Hydrate (DAB) solution. The sections were dehydrated and sealed, we assessed the area of the dermis stained by applying Image J image analysis software, which measured the dermal area from the top of the dermal papilla to the deepest part of the reticular dermis until adipose tissue or muscle formation.

***Statistical analysis***

Data were represented as the mean ± standard deviation (SD). Different asterisks on the bars indicated a statistically considerable difference in contrast with the designated control group (*P*<0.05). Under the premise that the sample data met the normal distribution, statistical significance was determined by one-way analysis of variance (ANOVA). In the case of homogeneity of variances, Bonferroni adjustment was performed; whereas variances were not homogeneous, the Dunnett T3 test was used for correction. When the measurement data did not conform to the normal distribution, the Kruskal-Wallis hr method in the non-parametric test method was applied for comparison between groups. All data were analyzed using SPSS 20 statistics software (SPSS, Inc., Endicott, NY, USA) or GraphPad Prism 8 software (GraphPad Software, San Diego, USA).

## Results

***(-)-α-Bis*** ***Increased cell viability in D-Gal-induced HSF cells***

**Figure 1:**
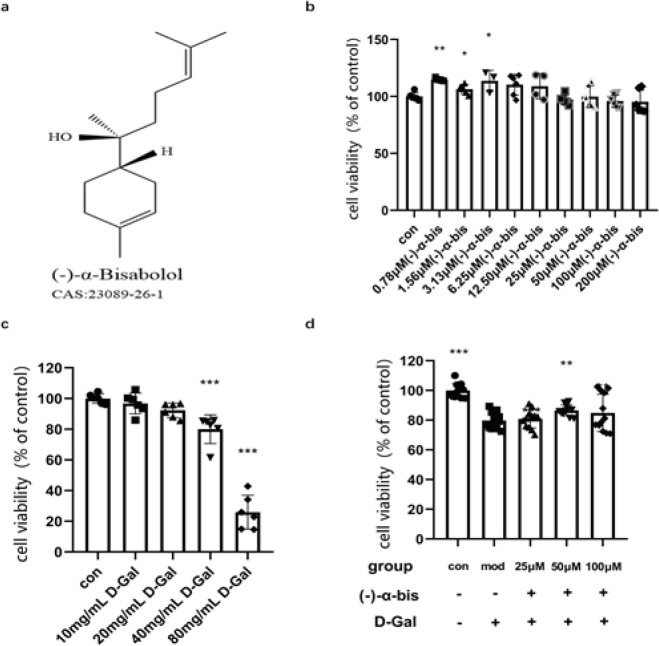
Effect of (-)-α-bis on the viability of HSF cells stimulated by D-Gal

The result of the cytotoxicity experiment on HSF cells treated with (-)-α-bis of different concentrations for 24 hr showed that (-)-α-bis did not significantly reduce the viability of HSF cells, suggesting that (-)-α-bis was a safe and non-toxic molecule (Figure 1b). We selected 25 μM, 50 μM, and 100 μM (-)-α-bis for follow-up experiments. HSF cells were stimulated by D-Gal at different concentrations for 24 hr, and the result of the MTT experiment showed that (-)-α-bis improved cell viability of HSF cells in a dose-dependent manner (Figure 1c). In this study, 40 mg/ml D-Gal concentration was selected for the next experiment (*P*<0.05). Subsequently, we observed whether (-)-α-bis of different concentrations had a protective effect on the survival rate in D-Gal-induced HSF cells. The result exhibited that 50 μM (-)-α-bis increased the survival rate of HSF cells under D-Gal stimulation (*P*<0.05) (Figure 1d).

***(-)-α-Bis reduced SA-β-gal expression in D-Gal-induced HSF cells***

**Figure 2:**
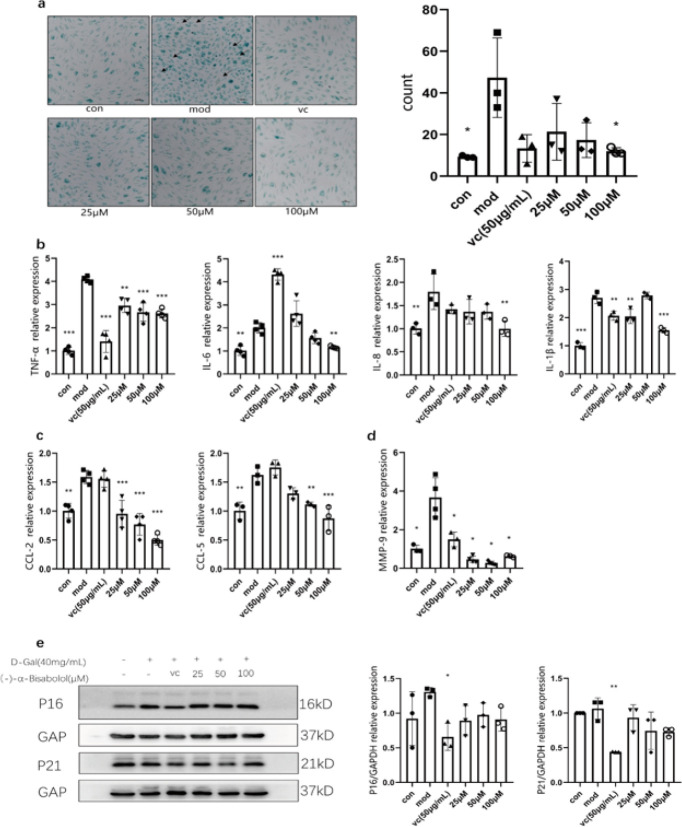
Effect of (-)-α-bis on D-Gal-induced relevant aging phenotypes in HSF cells

We investigated the effect of (-)-α-bis on SA-β-gal expression in HSF cells induced by D-Gal. It has been reported that SA-β-gal expression is remarkably increased in aging cell model systems (14). The possible effect of (-)-α-bis on SA-β-gal staining was measured in the present study. As seen in Figure 2a, cells treated with D-Gal (i.e., the mod group) exhibited more extensive and intense positive staining than cells of the control group, compared to the cells in the control group. In addition to the positive drug, vitamin C being great at precluding cells from becoming blue, (-)-α-bis (25 μM, 50 μM, and 100 μM) treatment groups had outstanding protective effects on D-Gal-induced aging. Aborative observation under the light microscope showed that D-Gal-induced senescent cells became flattened and vacuolated, and even some stress particles appeared, which rarely appeared in normal cells. However, the (-)-α-bis administration groups generally maintained a normal spindled state and fully spread.

***(-)-α-Bis ameliorated the SASP of D-Gal-induced HSF cells***

Intracellular signal transduction activated by DNA damage and cell cycle arrest indirectly stimulates the secretion of various proteins or factors in senescent cells. This is often referred to as SASP, which is often accompanied by cell senescence (15). The SASP of aging skin can be divided into many categories, such as inflammatory factors, chemokines, growth factors, matrix metalloproteinases (MMPs), etc (16). 

(-)-α-bis could down-regulate expression levels of TNF-α, IL-6, IL-8, IL-1β, CCL-2, CCL-5, and MMP-9 in D-Gal-stimulated HSF cells (Figure 2b, c, d). Cyclin-dependent kinase inhibitor (CKI) is an important signal molecule regulating cell proliferation. p53, p21, and p16 are the most representative indicators of cell cycle regulation commonly used to detect cell senium (17). As observed in Figure 2e, in contrast to the GAPDH, the expression of p16 and p21 was increased in the mod group.

The positive drug vitamin C could significantly improve the blue staining of HSF cells. In addition, vitamin C could significantly down-regulate the gene expression of TNF-α, IL-1β, and MMP-9 induced by D-Gal. Western blot results showed that vitamin C could significantly down-regulate the expression of p16 and p21.

***(-)-α-Bis meliorated the surface measurement of aging skin in vivo***

**Figure 3:**
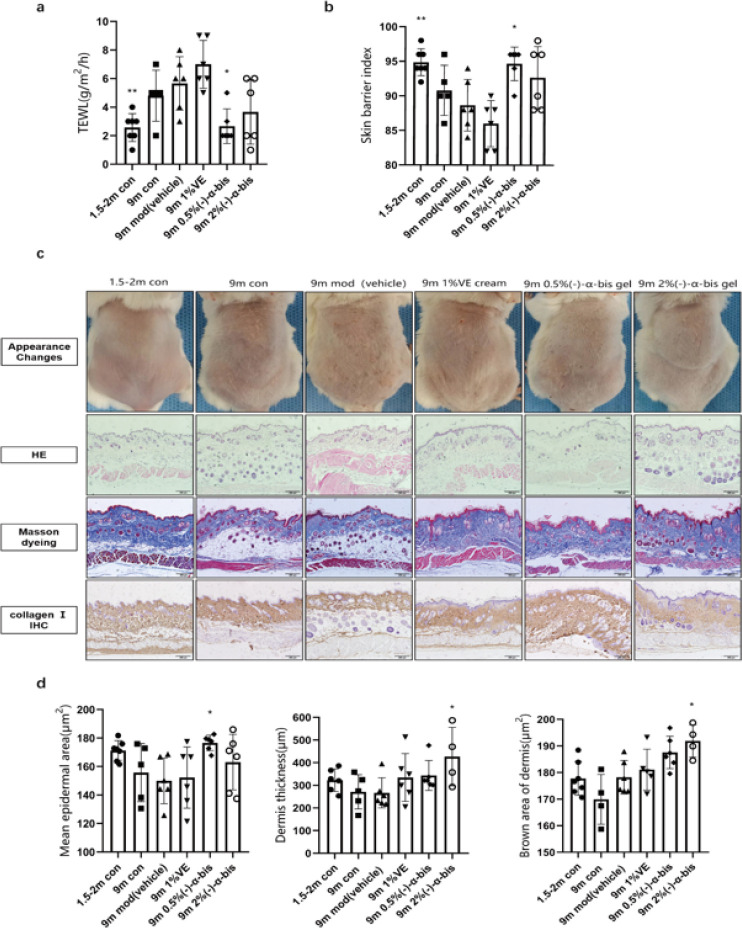
Effects of (-)-α-bis on related detection of aging skin in naturally aged 9 m BALB/c mice

To explore the effect of (-)-α-bis on natural aging skin appearance *in vivo*, we tested TEWL and skin barrier index in 9 m BALB/c mice using the gpskin barrier function meter. (-)-α-bis gel was applied to the skin on the back of mice for 30 days, twice a day, and a thin layer of gel was smeared to the dorsal skin of mice each time. The results of the gpskin barrier function measurement presented that compared with the 9 m mod group, the TEWL of the 9 m low dose group was considerably decreased, similar to that of the 1.5-2 m con group (*P*<0.05), while there was no obvious difference between 9 m high dose group and 9 m mod group (*P*>0.05) (Figure 3a). In contrast to the 9 m mod group, the skin barrier index was ascending in the 9 m low dose group (*P*<0.05), while there was no difference between the 9 m high dose group and the 9 m mod group (*P*>0.05) (Figure 3b). Representative photos of mice at the end of the treatment time proved that the skin of (-)-α-bis gel treatment groups was altered for the better (Figure 3c). The hair and skin on the back of the 9 m mod group were found to be drier, and the roughness degree of the skin was distinctly increased compared to the 9 m con group. These results indicated that (-)-α-bis treatment could improve skin smoothness and protect the aging skin barrier.

***(-)-α-Bis aggrandized epidermal and dermis thickness in vivo***

Epidermal thickness has been used as a mensurable parameter to evaluate skin aging (18). Thus, we valued the effect of (-)-α-bis gel administration on epidermal thickness in aged mice (Figure 3c,d). Hematoxylin and eosin staining expressed that the epidermis thickness of the 9 m mod group was thinner than other groups. The epidermal thickness of the 9 m low dose group (176.60 ± 5.42 μm) was nearly 1.1 times higher than that of the 9 m mod group (150.00 ± 16.05 μm), and (-)-α-bis increased the epidermal thickness (*P*<0.05) (Figure 3c). However, Masson staining can show the morphology and quantity of collagen fibers in the dermis simply and clearly, which is a conventional method to detect collagen fibers. Experimental results of Masson staining exhibited that the structures of skin tissues of the 1.5–2 m negative control group were complete with dense corrugation, uniform collagen arrangement, and fine fibers. The thickness of collagen fibers in the dermis was obviously thinner and the arrangement of collagen fibers was obviously loose, irregular, thick, curled, and stacked in the 9 m mod group. Compared with the 9 m mod group, the dermal thickness of skin tissues in (-)-α-bis treatment groups including the 9 m low dose group and 9 m high dose group increased, and collagen fibers were arranged more regularly (Figure 3c,d).

***(-)-α-Bis suppressed skin collagen degradation but inhibited the expression tendency of MMP-2 and MMP-3 in vivo***

**Figure 4:**
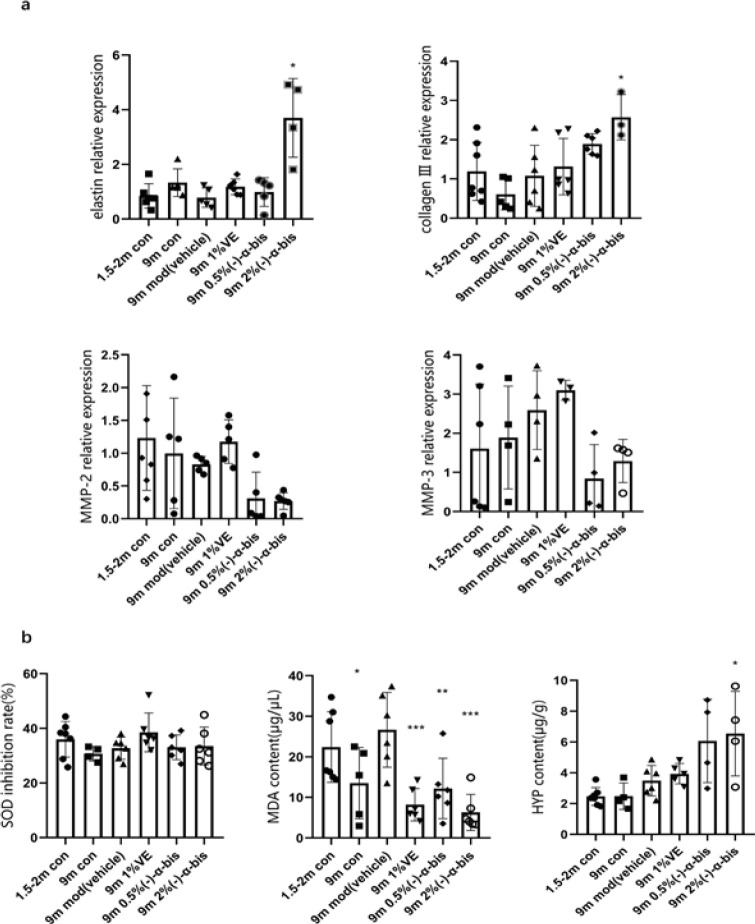
SOD inhibition rate, contents of MDA and HYP, and interrelated RT-qPCR indexes were detected in the dorsal skin of mice

As the skin ages, the collagen in the skin of mice degrades. To study the anti-aging effect of (-)-α-bis on the skin of aged mice, the expression of collagen Ⅰ was determined by immunohistochemical staining. The IHC results of collagen Ⅰ were as follows, compared with the 9 m mod group, the expression of collagen Ⅰ of (-)-α-bis high dose group in the dermis was increased, which could be observed by the reduction of the dermal brown area in the mod group (*P*<0.05). Topical application of the (-)-α-bis management groups averted this reduction (Figure 3c, d), indicating that (-)-α-bis suppressed degradation of collagen Ⅰ in naturally aged mice. To further verify the anti-decrepitude effect of (-)-α-bis on the skin, RT-qPCR was used to determine expression levels of elastin, collagen Ⅰ, MMP-2, and MMP-3 in the skin tissues of mice. The results represented that (-)-α-bis reduced the degradation of elastin and collagen Ⅰ in aged mice and inhibited the expression tendency of MMP-2 and MMP-3 in aged mice (Figure 4a).

***(-)-α-Bis reduced MDA and increased HYP content in vivo***

For further verifying the anti-aging effect of (-)-α-bis, SOD inhibition rate, MDA content and HYP content were detected in the back skin of mice. SOD can eliminate and reduce excessive free radicals in the human body, thus delaying aging (19). However, the result of the detection seemed to be unexpected. There was no trend in the 9 m mod group and (-)-α-bis intervention groups, but the result still made sense, probably because they did not induce a stress response in the mice (*P*>0.05) (Figure 4b). MDA level reflects the severe degree of free radical attack on the organism’s cells indirectly. As shown in Figure 4b (middle), MDA content was significantly increased in the 9 m mod group while being distinctly reduced in (-)-α-bis treatment groups (*P*<0.05). This indicated that (-)-α-bis could reduce the content of MDA in the skin of mice. The measurement of HYP in the skin can be also used as an indicator for screening drugs for antisenescence (20). The test result was shown in Figure 4b. Compared with the 9 m mod group, the 9 m high dose group exhibited an increased content, indicating that (-)-α-bis could increase HYP content in the skin of mice (*P*<0.05).

## Discussion

Delaying skin aging is extremely crucial for normal physiological function and mental health. (-)-α-bis is known to possess multifarious biological activities, including chemoprophylaxis, in particular, predecessors’ study proposes that (-)-α-bis reduces proinflammatory cytokine production and improves skin inflammation (21). However, prevenient data lack temporarily the anti-aging effect of (-)-α-bis on skin and mechanisms relative to changes. In this study, we investigated for the first time the anti-aging effect and possible potential molecular mechanism of (-)-α-bis in HSF cells. The intervention effect of (-)-α-bis on the skin of naturally aged 9-month-old BALB/c mice is also investigated.

(-)-α-bis reduces SA-β-gal expression and ameliorates the expression of TNF-α, IL-6, IL-8, IL-1β, CCL-2, CCL-5, and MMP-9 in SASP, and even lessens the tendency of cyclin p16 and p21 expression in HSF cells at the screened safe concentration range by the MTT assays. In the case of senescence of HSF cells induced by D-Gal, senescent cells exhibit morphological abnormalities, such as increased volume, multiplicative granules, enlarged nuclei, irregular shape, and apparent flattening when growing on solid surfaces. When SA-β-gal assays are performed at PH 6.0, blue-stained precipitates are observed in senescent HSF cells. The whole phenomenon is contributed by the specific overexpression and accumulation of endogenous lysosomal β-gal in senescent HSF cells and can be easily and reliably detected *in vitro*. Cells that express blue color are SA-β-gal positive, in consequence, the percentage of positive cell stained indicates senium specificity. Cellular senescence is frequently accompanied by production of SASP. SASP is composed of proinflammatory cytokines (TNF-α, IL-6, IL-8, and IL-1β), chemokines (CCL-2 and CCL-5), MMPs (MMP-9), and so on. SASP has the capacity to alter the cellular microenvironment of HSF cells, thereby inducing the senescence of other normal cells. For one thing, it can expedite the evil transformation of neighboring recipient cells, bringing about inflammation and diseases. For another, the immune system can be activated to remove senescent cells (22). IL-6 has been implicated in the senescence of keratinocytes, fibroblasts, and melanocytes caused by DNA injury (23). IL-6 and IL-8 are also relevant to a variety of physiological processes, inflammation included. IL-1 expression level is increased in decrepit fibroblasts and epithelial cells, and then combines with IL-1/Toll-like receptor to motivate NF-κB, therefore increasing the expression of SASP (24). Chemokines such as CCL-2 and CCL-5 and MMPs such as MMP-9 play key roles in regulating the migration of cells in damaged skin such as keratinocytes, fibroblasts, epithelial cells, and inflammatory cells by altering the damaged skin matrix (25). High expression of MMPs indicates accelerated degradation of extracellular matrix (ECM), resulting in natural and light aging of skin. Hence, to some extent, inhibiting or reducing secretion and expression of the SASP can help delay the aging of HSF cells and this experiment proves it. Cell senescence is mainly characterized by irreversible cell growth cycle arrest, which is principally due to the regulation of p16/RB and p53/p21 pathways. p16 and p21, as potential molecular targets, provide a new idea for the experimental research strategy of targeting skin aging to delay systemic aging (26). The regulation and changes of these two pathways may also be two theoretical signal transduction mechanisms conducing to the senescence of HSF cells (27). Therefore, it is found in this research that (-)-α-bis can delay the senescence of HSF cells by lessening the expression of SA-β-gal and SASP phenotypes, and its anti-senescence mechanism may be the reduced expression of key cyclin proteins p16 and p21 in p16/RB and p53/p21 pathways.

Furthermore, we demonstrate the ameliorative effect of (-)-α-bis on surface measurement of aged skin in naturally aged 9-month-old BALB/c mice. We use BALB/c mice at 1.5 to 2 months and 9 months of age as the aging model directly and successfully. No mice die during the whole process, and the aging model is stable and reliable. We utilize only male mice as an intracorporal model, but predecessors’ research has shown that the natural aging model is not affected by the estrous cycle (28). Therefore, we determine that topical application of (-)-α-bis gel could take a turn in the apparent skin condition of the back of naturally aging mice for the better. Moreover, aging skin may disrupt skin barrier function, leading to impaired skin barrier. Compared with the 9 m mod group, the TEWL level in the low-dose treatment group is visibly decreased after 30 days, and the TEWL level is inhibited by 0.5%(-)-α-bis gel treatment. The results of gpskin also show that the skin barrier index increased after (-)-α-bis administration. These two indexes have verified that (-)-α-bis has the effect of protecting the skin barrier in mice, but in the subsequent experimental studies, relevant skin barrier indexes such as skin barrier proteins can be added for detection. Histological research has revealed that aging skin is relative to declining epidermal thickness and alterations in connective tissue. Likewise, we observe that naturally aged BALB/c mice have thinner epidermis skin, whereas (-)-α-bis increases epidermal thickness (29). As opposed to UV-induced epidermal thickening (30), this is partly on account of a reduction in the proliferation and renewal capacity of keratinocytes and epidermal stem cells in the epidermis. It is a breakdown of the skin barrier and is possibly related to the loss of moisture from the skin. The decline in dermal thickness is the result of reduced fibroblast activity, decreased proliferation ability, and significant changes in dermal composition with age, which can be easily observed by Masson staining. However, dermal collagen fibers are mainly composed of collagen I and collagen III, so the content of collagen I in the dermis also decreases, which can be easily detected by the immunohistochemical method. In this research, the above internal model is utilized to demonstrate that (-)-α-bis can delay the senescence of HSF cells by reducing the expression of SA-β-gal and SASP factors. The mechanism of action in cell experiments is associated with the down-regulation of cyclin p16 and p21. Improved skin barrier function as well as SASP of skin is responsible for the delay of skin aging in naturally aged BALB/c mice in *in vitro*.

**Table 1:** RT-qPCR primer sequences

**Human**	**Forward primer(5′-3′)**	**Reverse primer (5′-3′)**
TNF-α	CCTCTCTCTAATCAGCCCTCTG	GAGGACCTGGGAGTAGATGAG
IL-6	ACTCACCTCTTCAGAACGAATTG	CCATCTTTGGAAGGTTCAGGTTG
IL-8	TTTTGCCAAGGAGTGCTAAAGA	AACCCTCTGCACCCAGTTTTC
IL-1β	ATGATGGCTTATTACAGTGGCAA	GTCGGAGATTCGTAGCTGGA
MMP-9	TCTATGGTCCTCGCCCTGAA	CATCGTCCACCGGACTCAAA
CCL-2	CAGCCAGATGCAATCAATGCC	TGGAATCCTGAACCCACTTCT
CCL-5	CCAGCAGTCGTCTTTGTCAC	CTCTGGGTTGGCACACACTT
GADPH	CTGGGCTACACTGAGCACC	AAGTGGTCGTTGAGGGCAATG

(a) Structural formula of (-)-α-bis. (b) Effect of (-)-α-bis on cytotoxicity in HSF cells. Cells were co-cultured with (-)-α-bis of numerous concentrations (25 μM, 50 μM, 100 μM, and so on) for 24 hr. Data were the mean ± SD of each independent experiment (n= 6, per group), *P<0.05, **P<0.01, ***P<0.001 vs the con group. (c) Effect of different concentrations of D-Gal on cell viability in HSF cells. Data were the mean ± SD of each independent experiment (n= 6, per group), *P<0.05, ** *P*<0.01, ***P<0.001 vs the normal group. (d) Protective action of (-)-α-bis on D-Gal-induced HSF cells. The data represented the values of mean ± SD (n= 12, per group), *P<0.05, **P<0.01, ***P<0.001 vs the D-Gal group. (-)-α-bis: (-)-α-Bisabolol; D-Gal: D-Galactose; HSF: Human skin fibroblasts

(a) HSF cells were pretreated with (-)-α-bis for 12 hr and then stimulated with 40 mg/ml D-Gal for 24 hr. The morphology of SA-β-gal staining in HSF cells was observed by a light microscope (× 200) where arrows pointed to particles. The cells were stained blue, and the data represented three independent experiments. The number of cells stained with SA-β-gal kit was shown on the right in Figure 2a, *P<0.05, **P<0.01, ***P<0.001 vs the mod group. (b) mRNAs of inflammatory factors TNF-α, IL-6, IL-8, and IL-1β in SASP were measured by RT-qPCR. Data were exhibited as the mean ± SD, as determined from three independent experiments. *P<0.05, **P<0.01, ***P<0.001 indicated a noteworthy difference from the mod group. (c) mRNAs of chemokines CCL-2 and CCL-5 in SASP were measured by RT-qPCR. Data analysis was the same as Figure 2b, *P<0.05, **P<0.01, ***P<0.001 vs the mod group. (d) mRNA of matrix metalloproteinase MMP-9 in SASP was measured by RT-qPCR. Data analysis was the same as Figure 2b, *P<0.05, **P<0.01, ***P<0.001 vs the mod group. (e) HSF cells were pretreated with (-)-α-bis for 12 hr and induced with 40 mg/ml D-Gal for 24 hr, then p16 and p21 protein levels were detected by immunoblot. Data analysis was the same as Figure 2b, *P<0.05, **P<0.01, ***P<0.001 vs the mod group.

(-)-α-bis: (-)-α-Bisabolol; CCL-2: Chemokine (C-C motif) ligand 2; CCL-5: C-C chemokine ligand 5; D-Gal: D-Galactose; HSF: Human skin fibroblasts; MMP-9: Matrix metalloproteinase-9; SA-β-Gal: Senescence-associated β-galactosidase; SASP: Senescence-as-sociated secretory phenotype

(a) After (-)-α-bis gel was applied to animals for 30 consecutive days, the TEWL of the dorsal skin of mice on the last day was calculated. *P<0.05, **P<0.01, ***P<0.001 vs 9 m mod group. (b) Skin barrier index was measured by the gpskin barrier function meter. *P<0.05, **P<0.01, ***P<0.001 vs 9 m mod group. (c) Changes in skin appearance, HE staining, Masson staining, and immunohistochemistry of collagen I in BALB/c mice treated with (-)-α-bis gel for 30 days were shown as follows. 9 m VE, 9 m low dose group, 9 m high dose group vs 9 m mod group. (d) From left to right, the statistics of average epidermal area, dermal thickness, and dermal brown area were revealed. The asterisk on the bars indicates a statistically considerable difference compared with the 9 m mod group. *P<0.05, **P<0.01, ***P<0.001 vs 9 m mod group.

(-)-α-bis: (-)-α-Bisabolol; TEWL: Trans epidermal water loss; VE: Vitamin E; 1.5–2 m: 1.5 to 2 months; 9 m: 9 months

(a) RT-qPCR was taken to detect the mRNA expression of elastin, collagen Ⅲ, MMP-2, and MMP-3. *P<0.05, **P<0.01, ***P<0.001 vs 9 m mod group. (b) From left to right, SOD inhibition rate, MDA content, and HYP content in back skin samples of BALB/c mice in turn. The asterisks on the bars indicate a statistically significant difference compared with the 9 m mod group. *P<0.05, **P<0.01, ***P<0.001 vs 9 m mod group.

HYP: Hydroxyproline; MDA: Malondialdehyde; MMP-2: Matrix metalloproteinase-2; MMP-3: Matrix metalloproteinase-3; SOD: Superoxide dismutase; VE: Vitamin E; 1.5–2 m: 1.5 to 2 months; 9 m: 9 months

**Figure 5:**
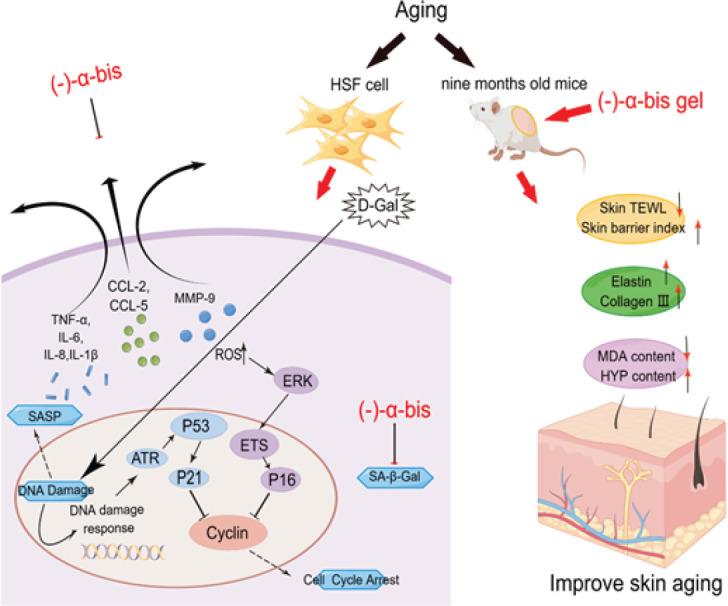
Simplified depiction of the proposed anti-skin aging mechanism of (-)-α-bis

(-)-α-bis can delay D-gal-induced HSF cell senescence by inhibiting the expression of SA-β-gal and SASP factors. Improved skin barrier function as well as SASP is responsible for the delay of skin aging in nine-month-old mice in the research. (-)-α-bis: (-)-α-Bisabolol; D-Gal: D-Galactose; HSF: Human skin fibroblasts; SA-β-Gal: Senescence-associated β-galactosidase; SASP: Senescence-as-sociated secretory phenotype

## Conclusion

(-)-α-Bis inhibits D-Gal-induced aging of HSF cells *in vitro* and inhibits aging skin in aged mice *in vivo*. Considering the biological function of (-)-α-bis, we suppose that (-)-α-bis may act as a potentially effective candidate drug for the treatment of aging in the skin. Our experiments indicate that p16 and p21 might be potential molecular targets for (-)-α-bis to inhibit D-Gal-induced cellular senescence (Figure 5).

## Data Availability

The raw measurements that support the findings of this study are openly available in figshare at http://doi.org/10.6084/m9.figshare.22040882, reference number: DOI: 10.6084/m9.figshare.22040882.
